# Psychological well-being and resilience experiences of educators teaching in juvenile correctional facilities: a scoping review

**DOI:** 10.3389/fpsyg.2025.1743604

**Published:** 2026-01-13

**Authors:** Innocent Sifelani, Wandile F. Tsabedze

**Affiliations:** Department of Psychology, University of South Africa, Pretoria, South Africa

**Keywords:** educator, experiences, juvenile correctional facilities, psychological well-being, resilience

## Abstract

**Introduction:**

Education is widely acknowledged as a cornerstone of rehabilitation for incarcerated youth. However, less attention has been paid to the specific realities faced by the educators who deliver it. These educators often operate under intense stress, experiencing burnout and emotional exhaustion while managing the complex and multifaceted needs of their students. This scoping review seeks to examine and synthesize psychological well-being and resilience experiences of educators working within juvenile correctional facilities.

**Methods:**

Our methodology was informed by the Joanna Briggs Institute (JBI) Manual for Evidence Synthesis. Relevant literature was sourced primarily from; ERIC, PsycINFO, Criminal Justice Abstracts with Full Text, Academic Search Complete, JSTOR, Google Scholar, Scopus, and Web of Science. The studies span an unspecified publication period and a systematic process guided the identification, selection, data extraction, and synthesis of findings.

**Results:**

This review examined a total of eight studies, comprising of seven empirical investigations and one conceptual paper. Notably, six out of the seven empirical studies were undertaken within the United States context. Four themes emerged from this review; challenges of teaching in correctional settings, psychological wellbeing, coping mechanisms, and resilience.

**Discussion:**

Educators in juvenile correctional settings work in environments fraught with numerous and complex challenges which adversely affect their psychological wellbeing. Despite these adversities, many educators demonstrated notable resilience cultivated through personal and social resources. Future studies should be conducted in low and middle income countries to better understand global variations and support systems.

## 1 Introduction

There is general agreement among legal scholars that the first distinct juvenile justice system was established in the United States in 1899, marked by the creation of the first juvenile court in Chicago (Zimring, 1999). Following this development, especially during the latter half of the 20th century, juvenile courts became a common feature across all jurisdictions in the United States and much of Western Europe. In Africa, the establishment of juvenile courts was largely a product of colonial influence (Odongo, 2004).

Juvenile detention facilities emerged alongside the establishment of the first juvenile courts in the late 19th century. These institutions were founded with a rehabilitative rather than punitive intent, aiming to support the development of delinquent youth into responsible and productive members of society (Roush, 1996). Similarly, (Pazé 2013) notes that contemporary approaches in juvenile criminal justice increasingly emphasize rehabilitation over punishment, focusing on the recovery and development of the young person. Within this framework, the role of educators becomes particularly vital as they support and guide minors in making sense of their life experiences, helping them to reflect, gain empowerment, and re-evaluate their future trajectories (Mastropasqua et al., 2020). However, the growing and complex needs of youth in juvenile justice facilities can place significant demands on staff (Jolivette et al., 2019). On the other hand, working with minors in juvenile justice settings presents educators with a range of challenges, stemming both from the complexity of the environment (Bouchard and Kunze, 2003; Muschitiello, 2019) and from organizational constraints (Accordini et al., 2015).

Access to formal education during incarceration can offer young people a meaningful pathway out of the criminal justice system (Davis et al., 2014). Ensuring that incarcerated youth receive a high-quality education while in correctional facilities can help mitigate some of the challenges they face during reintegration into society (Bozick et al., 2018). Furthermore, providing appropriate and responsive educational opportunities has the potential to enhance their academic performance, support positive behavioral development, and improve post-release outcomes (Houchins et al., 2017). Importantly, research has further demonstrated that when educators in such demanding contexts experience greater psychological well-being and resilience, they are better equipped to provide meaningful support to the youth in their care (Hwang et al., 2017).

Juvenile correctional centers constitute an exceptionally complex and demanding educational environment, markedly different from mainstream public school systems (Murphy, 2017; Reed, 2017). Educators in these institutions engage with highly vulnerable learners, many of whom grapple with intersecting challenges such as past trauma, neglect, abuse, and both diagnosed and undiagnosed learning difficulties or mental health conditions (Jolivette et al., 2019; Murphy, 2017; Baker, 2021). The teaching context is further complicated by stringent security protocols, frequent turnover in student populations, constrained instructional resources, and an overarching atmosphere of restriction all of which can hinder both teaching efficacy and learner engagement (Flores and Barahona-Lopez, 2020; Reed, 2017).

As a result, educators in these settings often encounter elevated levels of job-related stress, emotional exhaustion, and symptoms of secondary traumatic stress, contributing to persistent issues around staff retention and professional well-being (Bradshaw, 2024; Jolivette et al., 2019; Murphy, 2018; Baker, 2021). The psychological well-being and resilience of these educators are therefore not only central to their own occupational sustainability but also pivotal in enabling the transformative work they undertake with incarcerated youth (Bradshaw, 2024; Paoletti et al., 2023). Education delivered in juvenile justice settings plays a critical role in reducing recidivism and equipping young people with the academic competencies and life skills necessary for reintegration into society (Bradshaw, 2024; Flores and Barahona-Lopez, 2020). Yet, the intensity and complexity of this role exacerbated by the lack of specialized training, emotional support, and professional development frequently leave educators feeling professionally isolated, emotionally depleted, and underappreciated (Murphy, 2017, 2018; Reed, 2017).

While there is increasing acknowledgment of the vital role educators play in supporting the rehabilitation and reintegration of incarcerated youth, scholarly attention to their own psychological well-being and resilience remains limited (Bradshaw, 2024). A scoping review is methodologically appropriate for addressing this gap, as it facilitates a systematic and integrative examination of the breadth and depth of existing research. Specifically, this review allows for the mapping of current evidence, the delineation of conceptual and theoretical boundaries, and the critical analysis of how constructs such as psychological well-being and resilience are conceptualized and operationalized across diverse contexts (Arksey and O'Malley, 2005; Levac et al., 2010). Such an approach is particularly valuable in areas that are emergent, fragmented, or under-researched, where variations in terminology, methodological design, and disciplinary orientation often pose a threat in the synthesis of knowledge and the development of a coherent research agenda.

In accordance with guidance from the JBI Manual (Peters et al., 2020), a search of the literature was conducted across major databases including ERIC, PsycINFO, Criminal Justice Abstracts with Full Text, Academic Search Complete, JSTOR, Google Scholar, Scopus and Web of Science, Sabinet African Journals, HeinOnline, UNISA ETD: electronic theses and dissertations, Google and Google Scholar. This search revealed that no scoping reviews have yet been published on the topic. Consequently, the purpose of this scoping review is to identify and systematically map the existing body of peer-reviewed and gray literature on Psychological Well-Being and Resilience Experiences of Educators Teaching in Juvenile Correctional Facilities. The findings of this scoping review will enable the identification and examination of gaps in existing knowledge, which is crucial for guiding future research efforts.

### 1.1 Review question

What is the extent and nature of the literature regarding the psychological well-being and resilience experiences of educators working in juvenile correctional facilities?

### 1.2 Operational definitions

#### 1.2.1 Educators

Professional staff members whose primary role involves the delivery of academic and/or vocational curricula to incarcerated juveniles.

#### 1.2.2 Psychological well-being

It is defined through the lens of individuals' daily mental load, that is, specifically looking at how they navigate occupational stress, find meaning in job satisfaction, and manage the heavy emotional and cognitive burdens of the correctional environment, including the profound sense of social isolation that often accompanies this work.

#### 1.2.3 Resilience

In this review, resilience is defined as the internal resourcefulness that allows educators to maintain psychological integrity despite the taxing nature of correctional work. It encompasses the ability to absorb the emotional shocks inherent in secure settings and to find a path toward recovery and renewed professional purpose.

#### 1.2.4 Juvenile correctional facilities

These are state-operated or privately managed residential institutions that house individuals under the age of 21 pursuant to a judicial order.

### 1.3 Inclusion criteria

The inclusion criteria focused on studies that addressed the following aspects:

- Educators (*population*) currently teaching in juvenile correctional facilities as well as those who have previously taught in such settings and can reflect on their experiences.
- Educators' experiences of psychological well-being and resilience (*concept*) in relation to their teaching roles.
- Juvenile correctional facility schools (*context*).
- Sources of evidence from any existing literature (*types of evidence sources*).

### 1.4 Exclusion criteria

The exclusion criteria included;

- Non-teaching (e.g., correctional officers, healthcare workers, administrative personnel) personnel in juvenile correctional schools.
- Correctional facility educators working with adult incarcerated populations.
- Students, inmates, or detainees as the primary population.
- Studies that do not address psychological well-being or resilience directly or indirectly.
- Studies focusing only on academic outcomes, pedagogical methods, or curriculum development without linking to educator well-being or resilience.
- Protocols, conference abstracts, or posters without full-text articles.
- Studies not reported in English language.

## 2 Theoretical framework for the review

This review is guided by a conceptual framework that considers both personal and organizational influences on the psychological well-being and resilience of educators working within correctional education settings. At the individual level, the framework draws from Ryff's (1989) model of psychological well-being, which outlines key elements such as a sense of goal in life, control over the environment and positive interpersonal relationships (Harasim, 2018). These dimensions help explain how educators sustain their psychological balance amid the complex demands of their roles. The second level of the framework is informed by the Job Demands-Resources model, which views workplace outcomes as shaped by the interaction between pressures and available supports (Demerouti et al., 2001). By combining these two perspectives, the framework enables a structured approach to mapping evidence, allowing for the identification of both personal coping resources and institutional conditions that may support or hinder well-being and resilience in correctional education environments.

## 3 Methods

This scoping review study utilized a systematic approach as informed by the Joanna Briggs Institute (JBI) Manual for Evidence synthesis methodology (Peters et al., 2021). According to Peters et al. (2021), a scoping review is a type of evidence synthesis that has the objective of identifying and mapping relevant evidence that meets pre-determined inclusion criteria regarding the topic, field, context, concept or issue under review. In a similar vein, Tricco et al. (2016) emphasize that the primary purpose of a scoping review is to examine the scope of existing literature, map and synthesize available evidence, and guide future research directions. Accordingly, the scoping review approach in this study facilitated a comprehensive exploration and mapping of literature related to educators' psychological well-being and resilience across a wide range of sources. To promote consistency and transparency in reporting, this scoping review adhered to the Preferred Reporting Items for Systematic Reviews and Meta-Analyses Extension for Scoping Reviews (PRISMA-ScR) guidelines. To further enhance transparency, a predefined plan was developed outlining the objectives, methodology, and reporting approach of the review.

According to Peters et al. (2015), scoping reviews aim to map the breadth of available evidence on a given topic, regardless of methodological quality, and are particularly valuable for exploring emerging fields, clarifying key concepts, and identifying knowledge gaps. Consistent with this approach, several scholars have argued that scoping reviews do not require the critical appraisal of individual studies (Arksey and O'Malley, 2005; Brien et al., 2010; Peters et al., 2020). Accordingly, this review does not include a formal quality assessment of the reviewed studies. The exclusion of quality assessment allowed for a more inclusive exploration of the diverse experiences within juvenile correctional education without the constraints of a formal risk-of-bias assessment.

### 3.1 Search strategy

The literature search covered peer-reviewed studies and gray literature with unspecified publication period. The primary search strategy was developed by a librarian to ensure the identification of relevant studies across fourteen databases, namely, ERIC, PsycINFO, Criminal Justice Abstracts with Full Text, Academic Search Complete, JSTOR, Google Scholar, Scopus, Web of Science, Sabinet African Journals, HeinOnline, UNISA ETD: electronic theses and dissertations, Google and Google Scholar. The authors then moved relevant extracted studies to RefWorks software for storage and citation management.

In conformity with guidelines for all JBI types of reviews, a three search strategy was used. First, the reviewers conducted an initial limited search of two appropriate online databases relevant to the topic, namely Google Scholar and ERIC. This first step enabled the identification of key words and phrases to inform the final search strategy. Second, a search making use of identified key words across all included data bases was conducted. Third, an examination of the reference lists from the included full-text sources was conducted.

The search strategy was structured around three core constructs: “juvenile educators” as the *population*, “psychological well-being and resilience” as the *central concept*, and “juvenile correctional facility schools” as the *context*. Each construct was expanded using a comprehensive list of related synonyms to ensure a broad and inclusive search. The authors, in collaboration with a librarian, applied a consistent Boolean search strategy incorporating truncation which was tailored to accommodate the unique configurations of each database's search engine (see Appendix A). Google Scholar served as the primary source for gray literature, where the same three constructs guided the identification of relevant studies. The literature search was conducted between May and June 2025, and one initial filter was applied: exclusion of non-human studies.

### 3.2 Screening and selection

A total of 630 references were initially imported into the RefWorks software for storage and citation management. The RefWorks tool identified and removed 88 duplicate entries, leaving 542 references. These were then transferred to Covidence to facilitate the scoping review process. Covidence identified and removed an additional 14 duplicates, resulting in 528 references for title and abstract screening.

Following the screening of titles and abstracts, 33 references were selected for full-text review in Covidence. The reviewers conducted the full-text screening based on predefined inclusion and exclusion criteria. A total of 25 studies were excluded for the following reasons: seven did not focus on juvenile educators; ten addressed challenges faced by educators but did not explore psychological well-being or resilience; three were set in non-juvenile correctional contexts; and five full texts were unavailable.

**Figure 1:**
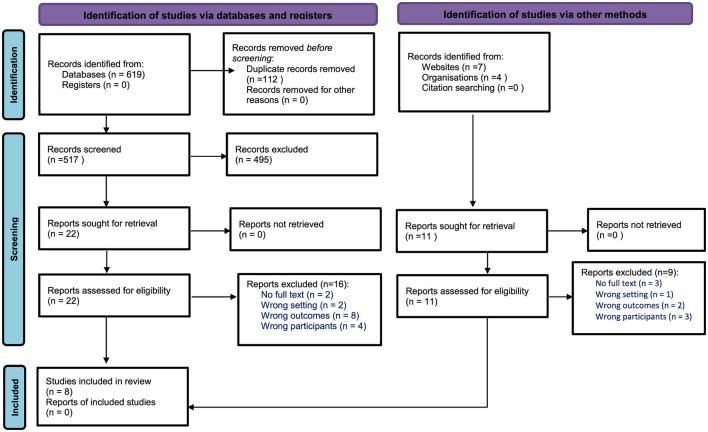
PRISMA flow diagram: screening of references.

Ultimately, 8 studies met the inclusion criteria and were retained for data extraction and analysis. The study selection process is summarized in Figure 1 via a PRISMA flow diagram.

### 3.3 Data charting, synthesis, and analysis

Data charting was conducted as an iterative and systematic process aimed at extracting key information from each included study (Levac et al., 2010). Reviewers independently and manually charted data using a standardized extraction form developed in accordance with the JBI Manual for Evidence Synthesis (Peters et al., 2021). The charted information included details such as author(s), year of publication, country of study, population characteristics, study aims, research design, data collection methods, and key findings. To ensure consistency and accuracy, data extraction was conducted independently by the reviewers and finalized through consensus-based discussion.

A descriptive qualitative content analysis was conducted, starting with data familiarization. Reviewers carefully analyzed the extracted data, focusing specifically on the results and discussion sections of the included studies. This was followed by open coding, in which segments of text are labeled with short descriptors that capture the core idea or experience being conveyed.

Once coding was complete, similar codes were grouped into broader categories that reflect commonalities across the data. These categories were then examined for higher-level patterns and synthesized into overarching themes that provided insight into the phenomena under investigation, in this case, the psychological well-being and resilience of educators in juvenile correctional settings. Eventually, findings were reported descriptively rather than analytically (see JBI Manual p. 453). A summary of included studies is shown in Table 1.

**Table 1:** Overview of studies included in the review.

**Study ID**	**Author and year**	**Title**	**Participants**	**Method**	**Key findings**
Study 1, USA	Baker (2021)	Teacher Wellbeing in a Juvenile Detention Facility.	2 Educators	Qualitative: Autobiography and biography (interview)	*Correctional environment and educator well-being:* • Teaching in juvenile detention centers is highly stressful, leading to challenges like Secondary Trauma Stress (STS), burnout, and emotional tension among teachers.• *Coping and resilience-building factors:* • Mindfulness practices and utilization of knowledge on Polyvagal Theory and Integral Theories. • Personal characteristics such as altruism and student-centeredness facilitated the ability to cope and stay resilient.
Study 2, USA	Bradshaw (2024)	Resilience of teaching juveniles: A phenomenological study in juvenile corrections facilities.	13 Educators	Qualitative: hermeneutic phenomenological study (interviews, exhibit scenarios and journal reflection)	*Corrections environment and educator well-being:* • A challenging environment characterized by threats to teacher safety posed by students, class disruptions and unavailability of other corrections staff when situations arose. • Experiences of burnout and other emotional challenges were reported.• *Coping and resilience building factors:* • Resilience was fostered by reflecting on their calling for the job, their belief in a higher power, a strong desire to help their students, practicing self-care and personal characteristics such as inner strength.
Study 3, USA	(Flores and Barahona-Lopez 2020)	“I am in a constant struggle:” The challenges of providing instruction to incarcerated youth in southern California.	15 educators	Qualitative: Semi-structured interviews and classroom observations	*Corrections environment and educator well-being:* • Student challenges included significant educational gaps making it difficult to align with the standardized curriculum • Unique and challenging corrections environment led to teachers' emotional labor, feeling overwhelmed and uneasy. • Experiences of being emotionally, psychologically and spiritually drained were highlighted. • Educators found teaching young women particularly challenging, perceiving them as “more emotional, needy and held more 'grudges' compared to young men, and sometimes exhibit “hostile” attitudes.• *Coping and resilience* • Strong sense of purpose and connection with students. • Love for the students propelled the educators to persevere.
Study 4	Jolivette et al. (2019)	Embedding Staff Self-Care into the MTSS Framework for Those Working in Juvenile Correctional Facilities.	Not applicable	Conceptual paper that synthesizes existing literature on stress, burnout, and self-care in juvenile correctional settings	*Corrections environment and educator well-being:* • Educators indicated experiences of stress, secondary traumatic stress and burnout as a result of challenging corrections setting and the nature of youths being served. • Juveniles have comorbid mental health needs, challenging behaviors, are traumatized and have educational disabilities. • The experienced challenges led to adverse impact on life-work balance as the affected educators think of the students even at home.• *Coping strategies:* • Self-care practices such as mindfulness exercises and wellness programs.
Study 5, USA	Murphy (2017)	“I'm Going to Need a Change…”: Understanding a Teacher's Experiences in a Juvenile Corrections School.	1 Educator	Qualitative: Interview	*Educator well-being:* • Isolation and stagnation in his work • Experiences of being bored were highlighted. • Frequent mention of concerns about becoming bored and needing a “different type of challenge”• *Coping and resilience:* • Personal characteristics and skills acted as resilience building factors
Study 6, USA	Murphy (2018)	Should I stay or should I Go? Teachers' Commitment to their Work in Juvenile Corrections schools.	5 Educators	Qualitative: Interviews	*Corrections environment and educator well-being:* • Challenging corrections environment led to experiences of (cognitive) dissonance, dismay, frustration, stress, and feeling an overwhelming sense of professional failure. • Stifling sensation also experienced. • Unmet/unrealistic expectations led to frustration resulting in contemplation to quit the job.• *Coping and resilience:* • Resilience building factors included; educator disposition, utilization of social capital, previous experience to maneuver around met challenges, anticipated positive student outcomes, low expectations
Study 7, Italy	Paoletti et al. (2023)	Training Spherical Resilience in Educators of the Juvenile Justice System during Pandemic.	30 Educators	Quantitative: Psychometric instruments	*Coping and resilience:* • Self-efficacy in managing emotions and positive attitude. • Problem solving skills being building blocks of resilience
Study 8, USA	Reed (2017)	Teach (-er/-ing) In Detention: The Experiences of Teachers in Juvenile Detention Facilities.	4 Educators	Qualitative: interviews	*Corrections environment and educator well-being:* • Unique (diverse characteristics and complex histories of the youth, limitations imposed by facility procedures, safety and security requirements, resource availability, physical structure, high student mobility, and varied lengths of stay) prison environment lead to experiences of isolation.• *Coping and resilience:* • Becoming flexible and content about the reality on the ground.

## 4 Findings

### 4.1 Study characteristics

Based on the stated aims of the eight studies reviewed, four studies (Baker, 2021; Murphy, 2017, 2018; Reed, 2017) concentrated on exploring teachers' lived experiences and working conditions within juvenile correctional facilities. Two studies (Bradshaw, 2024; Paoletti et al., 2023) specifically examined aspects of teacher resilience and psychological well-being in correctional settings. One study by (Flores and Barahona-Lopez 2020) focused on the unique challenges associated with delivering mandatory educational instruction in youth correctional systems. Finally, the study by Jolivette et al. (2019) outlined how the tiered framework of the Multi-Tiered System of Supports (MTSS) can be applied to support staff self-care, incorporating targeted strategies drawn from research on stress and burnout.

This review draws on seven empirical studies, six of which were conducted in the United States and one in Italy. In addition, one conceptual paper was included, which outlines a framework for addressing self-care needs among staff in correctional settings. All the studies were published within the past decade (2017–2024). Of the seven empirical studies reviewed, six employed qualitative research designs, primarily using interviews as the main method of data collection (Bradshaw, 2024; Flores and Barahona-Lopez, 2020; Murphy, 2017, 2018; Reed, 2017). One qualitative study (Baker, 2021) adopted an autobiographical and biographical narrative approach. The sole quantitative study (Paoletti et al., 2023) utilized self-report questionnaires to gather data.

Participant numbers in the qualitative studies varied, ranging from one to fifteen individuals. Specifically, Baker (2021) involved 2 participants; Bradshaw (2024) included 13 participants; (Flores and Barahona-Lopez 2020) engaged 15 participants; Murphy (2017) involved a single participant; Murphy (2018) included 5 participants; and Reed (2017) worked with 4 participants. In contrast, the quantitative study conducted by Paoletti et al. (2023) had a sample of 30 participants.

### 4.2 Study themes

This scoping review identified four themes which are presented in Table 2.

**Table 2:** Themes and sub-themes.

**Emerged themes**	**Sub-themes**
1. Challenges of teaching in correctional settings	• Volatile and emotional intense environment • Structural and institutional constraints • Lack of autonomy and support • Curriculum misalignment • Nature of students
2. Psychological well-being	• Emotional and Psychological Burden • Social isolation
3. Coping mechanisms	• Self-care practices • Social capital
4. Resilience	• Moral commitment and altruism • Background and early life experiences • Self-efficacy • Reality consciousness and realistic expectations • Belief system and culture

#### 4.2.1 Theme 1: challenges of teaching in correctional settings

The theme's focus is on challenges of teaching in correctional settings. Educators in juvenile correctional facilities work within spaces characterized with students of complex and varied needs. Furthermore, teachers are expected to navigate the complicated prison environment. Therefore, to truly comprehend the educators' psychological well-being and resilience, it is crucial to understand their lived experiences within the specific context of these challenging settings.

##### 4.2.1.1 Sub-theme 1: volatile and emotional intense environment

To offer insight into the nature of juvenile correctional facilities, the following vignette which has been drawn from Baker's (2021) study, illustrates a real-life scenario within this setting:

I want to share a snapshot from one afternoon that shows how a simple comment can unexpectedly spark chaos in the classroom. The students were quietly working on their assignments when the school liaison came in and had us all move to a different room. We ended up in the Art room, which had colorful paintings on the walls.
Not long after settling in, one student commented on how much she loved the blue paintings. I added that I thought the orange ones were bright and cheerful. She replied that she didn't like the red, and I simply said, “Ok then.” That's when things escalated.
Another student overheard her dislike of red and immediately questioned her by asking if she was a Crip. The first student confirmed that she was, even mentioning her “Big Homey.” Suddenly, students around the room started declaring their gang affiliations and titles. Then, another student burst into the conversation, furious. She shouted that she knew the first girl's gang leader and blamed him for hurting her brother. The anger on her face, her clenched fists, and the way her whole body tensed, I could see she was ready to fight.
The room filled with tension, and I felt my anxiety shoot up. I quickly signaled for a juvenile counselor to intervene. In seconds, desks were flying and the shouting got so loud my ears rang. Relying on my past experiences, I moved out of the way and ran to get more help. The counselor managed to remove the student who had started the argument, her hands behind her back.
My heart was pounding, not because I was afraid for myself but because I was worried about the girls. These kinds of outbursts can leave students physically hurt and emotionally scarred. Even though teachers aren't usually the target of the violence, we still carry the emotional toll. It's emotionally draining and distressing to witness such raw conflict unfold in front of you. (p. 15)

The above vignette portrays the juvenile correctional setting as a highly volatile and emotionally intense environment, where situations can escalate quickly and unpredictably. Baker (2021) further notes that teachers reported facing an emotionally charged environment where mental health and behavioral disorders among students were the norm, not the exception. It is therefore extremely fearful being around incarcerated juveniles (Baker, 2021), particularly because educators are at times subjected to verbal assaults by the students (Bradshaw, 2024). According to Bradshaw (2024), the persistent safety concerns expressed by teachers regarding their interactions with students contribute to making the correctional environment emotionally intense and psychologically taxing.

##### 4.2.1.2 Sub-theme 2: structural and institutional constraints

Murphy (2018) found that teachers often experienced an overwhelming sense of professional failure due to lack of institutional support, a stifling school environment, and nonexistent educational standards. (Flores and Barahona-Lopez 2020) weigh in by noting that the structure of correctional facilities presents unique challenges that hinder effective teaching and learning. According to Murphy (2018), teachers felt increasingly alienated and disengaged, reporting that even their efforts to improve outcomes were frustrated by unsupportive leadership and indifferent security staff. Research has further shown that correctional personnel often regard teachers with dislike, perceiving them as excessively compassionate or empathetic toward incarcerated juveniles (Zabel and Nigro, 2007). This perception can lead to behaviors that undermine the educational process, including the harassment of educators such as deliberately obstructing their access to classrooms or instructional spaces.

##### 4.2.1.3 Sub-theme 3: lack of autonomy and support

Reed (2017) and Bradshaw (2024) described how safety concerns and security procedures severely limit the autonomy of teachers and affect their instructional capacity. For instance, the need to adhere to correctional protocols often interferes with student engagement and continuity in teaching, particularly because of high student mobility and erratic lengths of stay in facilities.

##### 4.2.1.4 Sub-theme 4: curriculum misalignment

(Flores and Barahona-Lopez 2020) noted how the misalignment between the standardized curriculum and students' significant educational gaps created unrealistic instructional expectations. In addition to curriculum misalignment, teaching in correctional settings often involves working with students who face numerous educational difficulties, many of which are intensified by the nature of incarceration environment (Costelloe and Langelid, 2011). A study by (Flores and Barahona-Lopez 2020) found that incarcerated juveniles typically have low levels of educational attainment, including a significant number diagnosed with or suspected of having learning disabilities. Consequently, educators reported that teaching students with such gaps was particularly challenging as the prescribed curriculum often did not align with the students' actual educational levels.

Evidently, it has been established that the unique structural and institutional conditions within juvenile correctional centers place considerable strain on the psychological well-being of educators working in these settings (Flores and Barahona-Lopez, 2020).

##### 4.2.1.5 Sub-theme 5: nature of students

The corrections environment is marked by the presence of students who have experienced trauma and who also exhibit mental, emotional and behavioral disorders (Baker, 2021; Jolivette et al., 2019). Murphy (2018) reinforces this view by noting that incarcerated juvenile students represent a particularly challenging population to work with.

#### 4.2.2 Theme 2: psychological well-being

A recurrent theme across the studies is the intense emotional and psychological burden experienced by educators working in juvenile correctional centers. The teaching environment is consistently described as highly stressful, often marked by burnout, emotional exhaustion, and secondary traumatic stress (STS).

##### 4.2.2.1 Sub-theme 1: emotional and psychological burden

In Baker's (2021) autobiographical narrative inquiry, participants disclosed severe stress-related conditions, including high blood pressure and fear of physical harm, resulting from their interactions with emotionally unstable or violent students. Similarly, (Flores and Barahona-Lopez 2020) underscored the emotional toll on teachers, who described their work as “psychologically draining” and even “spiritually exhausting,” particularly when teaching incarcerated young women, perceived as more emotionally complex and challenging than their male counterparts.

Jolivette et al. (2019) added a broader conceptual framing, identifying that educators experience STS due to their constant engagement with trauma-exposed youth. As a result, the cumulative exposure to such emotionally intense conditions leads to feelings of helplessness, over identification with students' trauma, and difficulty separating work from personal life.

Murphy (2018) also highlighted feelings of cognitive dissonance, professional failure, and frustration among teachers who struggled to reconcile their expectations with the grim reality of correctional schooling. These findings reveal the intense mental burden educators face when attempting to teach in environments devoid of adequate institutional support, further compounded by the oppressive correctional climate.

##### 4.2.2.2 Sub-theme 2: social isolation

Another challenge faced by teachers in juvenile correctional facilities is a pervasive sense of isolation (Murphy, 2017). This feeling is often intensified by the limited availability of resources for educators working with incarcerated youth, as well as the absence of other correctional staff during critical situations (Bradshaw, 2024; Murphy, 2018; Reed, 2017).

#### 4.2.3 Theme 3: coping mechanisms

##### 4.2.3.1 Sub-theme 1: self-care practices

Self-care strategies including therapy services, prayer, mindfulness practices, taking time off from work and journaling were used as coping mechanisms and subsequently promoted desirable mental health of educators (Baker, 2021; Bradshaw, 2024; Jolivette et al., 2019). Among other recommendations, the study by (Flores and Barahona-Lopez 2020) highlighted the need for self-care practices as a measure to lessen the difficulties that corrections teachers experience.

##### 4.2.3.2 Sub-theme 2: social capital

Embracing the idea of seeking support from others has proven to be an effective coping strategy. Engaging in conversations with colleagues about shared challenges emerged as a valuable means of managing and navigating difficulties (Bradshaw, 2024). Additionally, Murphy (2018) indicated that a supportive work environment, characterized by collegial collaboration and encouragement, serves as a valuable coping resource for teachers working in juvenile correctional schools. According to Bradshaw (2024), having a support network helped the teacher participants to avoid feelings of isolation and insecurity when faced with challenges.

Relatedly, the establishment of meaningful relationships with students appears to foster mutual respect between educators and learners, while also contributing to the reduction of students' anger and enhancing their willingness to engage in classroom activities. According to Bradshaw (2024), many educators observed that when students felt genuinely seen, heard, and valued, their disruptive behaviors decreased, and their openness to learning increased. It is therefore ascertained that educators established relationships with students in an effort to cope with their disruptive behaviors.

#### 4.2.4 Theme 4: resilience

Despite the overwhelming challenges, the reviewed studies consistently found that educators possess or develop remarkable resilience. Their ability to adapt, maintain hope, and continue engaging with incarcerated youth reflects a complex interplay of personal, and spiritual, factors.

##### 4.2.4.1 Sub-theme 1: moral commitment and altruism

Moral commitment, selflessness and a philanthropic spirit have been identified as key factors that contribute to the development of resilience. Despite overwhelming challenges, most educators expressed strong loyalty to their students and a belief in the power of education to change lives. Many teachers maintained their roles because they viewed students not as criminals but as individuals worthy of care, growth, and opportunity (Baker, 2021; Bradshaw, 2024; Flores and Barahona-Lopez, 2020). The educators' narratives consistently reflected compassion, empathy, and a sense of justice. For example, one teacher stated, “It is them and their lives and their stories that keep me coming back” (Flores and Barahona-Lopez, 2020). Evidently, a deep love for both the profession and the students played a significant role in strengthening educators' resolve to persevere (Bradshaw, 2024).

Educators who identified strongly with their calling whether spiritually, ideologically, or personally, were more likely to stay in the profession and find meaning in their work. Bradshaw (2024) and Baker (2021) emphasized that moral purpose was not simply abstract idealism; it functioned as a daily motivator and resilience resource. It helped teachers to process trauma, transcend institutional failings, and maintain emotional engagement.

##### 4.2.4.2 Sub-theme 2: background and early life experiences

According to Murphy (2018), the vast background experience as a teacher acts as a protective factor to persevere and remain within the corrections education space. On another hand, some teachers developed resilience through early life experiences or childhood adversity that taught them persistence and emotional regulation. Illustratively, a participant in Baker's (2021) study revealed that her ability to persevere in the face of the demanding correctional environment was shaped by her strong Jamaican upbringing and her father's influence. In some cases, early life experiences served as foundational elements in the development of resilience fortitude (Bradshaw, 2024).

##### 4.2.4.3 Sub-theme 3: self-efficacy

Self-efficacy, which is the belief in one's ability to accomplish a specific task or goal, was identified as a key factor in fostering resilience. Paoletti et al. (2023) contributed a quantitative perspective, identifying self-efficacy, emotion regulation, and a positive outlook as key predictors of resilience. In concurrence, findings from Bradshaw's (2024) study revealed that the participants demonstrated characteristics of inner strength, self-efficacy, a positive outlook, and a refusal to give up.

##### 4.2.4.4 Sub-theme 4: reality consciousness and realistic expectations

Murphy (2017) emphasized realistic expectations on the nature of corrections schools as a foundation of resilience. In his 2018 study, Murphy further emphasized the importance of low expectations as a resilience strategy and not as a form of surrender but as a buffer against burnout. Reed (2017) observed that educators' acceptance and contentment with the realities of their working environment contributed to their resilience.

##### 4.2.4.5 Sub-theme 5: belief system

In Bradshaw's (2024) study, religion and faith emerged as a significant theme, with participants attributing their sense of calling and personal resilience to God or a higher power. Moreover, participants indicated that their relationship with God provided them with both guidance and the motivation to persevere, even during particularly challenging times. It is evident that teachers often drew strength from their spiritual beliefs-specifically the belief that their work was a “calling.”

## 5 Discussions

This scoping review set out to explore the existing literature on the psychological well-being and resilience of educators working in juvenile correctional centers. The review was particularly guided by an intention to illuminate insights that could inform realities and experiences of educators in low and middle income countries (LMICs), with a specific focus on the African context. The findings of this review highlight critical gaps in the current body of scholarship, both in terms of geographical representation and thematic focus.

One of the most noticeable patterns that emerged from this review is the geographical concentration of the literature. 85% of the reviewed empirical studies were conducted in the United States of America (Baker, 2021; Bradshaw, 2024; Flores and Barahona-Lopez, 2020; Murphy, 2018; Reed, 2017), with only a single study located in Italy (Paoletti et al., 2023). No studies were found from African countries, or from other continents such as Asia, South America. Drawing on Polit and Beck's (2010) assertion that the primary aim of most qualitative research is not to achieve generalizability, the overrepresentation of USA based research limits the global applicability of existing findings, as they are shaped by a particular socio-cultural and institutional framework unique to American correctional education systems.

The absence of research from the African continent is particularly concerning. Given the stark differences in economic resources, cultural norms, correctional philosophies, and educational infrastructure (Quainoo, 2023) between African nations and countries like the USA, this lack of localized evidence leaves a critical void. The experiences of educators from LMICs' juvenile correctional centers may differ significantly from their counterparts elsewhere due to distinct systemic constraints, socio-political dynamics, and cultural understandings of mental health and education. The current lack of African studies, therefore, underscores the urgent need for context-specific research that captures the voices and realities of African educators working within correctional facilities.

A second notable observation from this review is the thematic narrowness of existing literature. While many studies examined the challenges faced by educators in juvenile correctional centers, very few studies (Bradshaw, 2024; Paoletti et al., 2023) focused exclusively and directly on psychological well-being and resilience as primary constructs. The psychological and emotional experiences of educators were often treated as secondary findings by being implied within broader discussions of teaching conditions, or institutional challenges. This suggests that the “human element” of teaching has been marginalized in favor of structural or administrative data. When the human element is missing from the literature, the academic understanding of the field remains incomplete and skewed.

The majority of reviewed studies lacked a systematic or theoretical engagement with the concepts of psychological well-being or resilience. This lack of conceptual clarity also points to an absence of uniformity in how resilience and well-being are defined, measured, or discussed in the literature. Although some studies did not explicitly present coping strategies and resilience as interchangeable, their empirical use reflected an implied conflation of the constructs (Bradshaw, 2024), pointing to inconsistencies in conceptual clarity. As a result, our understanding of how educators cope, adapt, or thrive in correctional settings remains fragmented and underdeveloped. Without focused attention on these dimensions, opportunities to develop effective, evidence-based interventions and supports for educators are significantly constrained.

Although the assessment of methodological quality falls outside the scope of this scoping review, the inclusion of evidence of varying quality introduces certain limitations. For instance, the apparent prominence of specific themes or concepts may reflect the abundance of exploratory or descriptive studies in the literature, rather than their consistent confirmation across methodologically robust research.

Our mapping of the literature indicates a strong emphasis on qualitative approaches which are well suited to capturing rich, in-depth accounts of educators' well-being and professional commitment (Baker, 2021; Murphy, 2017; Reed, 2017). At the same time, these studies are frequently conducted within single sites or with small samples, which increase the likelihood of context-specific bias and limit the transferability of findings.

In addition, although some studies examined targeted resilience-building interventions (Paoletti et al., 2023), the lack of quality appraisal means we could not evaluate the rigor of their design precluded an assessment of their methodological rigor. Consequently, conclusions about the effectiveness of strategies to enhance well-being should be interpreted with caution. The findings of this review are therefore best understood as an overview of what has been explored and how educators' experiences have been described, rather than as a definitive evidence base for the implementation of new well-being initiatives.

Acknowledging the constraints previously identified, we sought to provide additional context by ensuring that our data extraction specifically captured details on study design, sample size, and type of setting for all included articles. Including these details offered some insight into the potential generalizability of the findings.

Ultimately, the main implication of this methodological choice (exclusion of quality assessment for included studies) is the need for further research. We strongly recommend that future studies conduct systematic reviews, allowing for a rigorous critical appraisal to assess the reliability and validity of the evidence.

## 6 Strengths and limitations

The search strategy was strengthened through consultation with an experienced academic librarian, ensuring methodological rigor and the inclusion of a broad and relevant range of studies. One limitation of this review is the exclusion of studies published in non-English language. This language restriction may have led to the omission of relevant research in non-English speaking contexts, thereby potentially narrowing the global applicability and cultural diversity of the findings.

## 7 Conclusions

Four themes emerged from this review; challenges of teaching correctional settings, psychological well-being, coping mechanisms and resilience. Educators in juvenile correctional settings work in environments fraught with numerous and complex challenges which adversely affect their psychological well-being. Despite these adversities, many educators demonstrated notable resilience, cultivated through personal and social resources. Key resilience enablers included a strong sense of purpose and altruism, spiritual grounding, and the strategic use of self-care techniques such as mindfulness. The value of social capital such as collegial support, therapy, and professional networks was emphasized as vital for sustaining psychological well-being.

Importantly, several studies underscored how personal histories and experiences, and realistic expectations shaped coping capacities. Teachers who maintained a positive outlook and found meaning in small student successes reported higher job satisfaction and lower levels of disillusionment. Finally, the correctional setting itself plays a critical role. Poor administrative support, inadequate leadership, and institutional rigidity were identified as major barriers to professional fulfillment and instructional efficacy.

## 8 Practical implications

The findings of this review carry significant implications for research, training, support programs, and policy in low and middle income countries (LMICs).

### 8.1 Future research

First and foremost, the findings call attention to the invisibility of LMICs' educators in global correctional education discourse. Without empirical data that reflects their voices, experiences, and contexts, the psychological needs of these educators are unlikely to be understood or addressed in meaningful ways. Future research in LMICs should therefore aim to fill this critical gap by conducting primary studies that explicitly examine the psychological well-being and resilience of teachers in juvenile correctional centers.

Furthermore, scholars should consider exploring resilience not solely as an individual trait but as an ecological construct influenced by systems, relationships, and structures. In doing so, future studies can contribute to a more holistic and humanizing understanding of what it means to teach, survive, and possibly thrive within the walls of juvenile correctional centers.

### 8.2 Trauma-informed pedagogy training

Educational authorities should move beyond general orientation and implement mandatory training focused on secondary traumatic stress and classroom management within volatile, high-intensity environments. This training must address the unique “nature of students” in correctional facilities to bridge the current gap in curriculum alignment.

### 8.3 Support programs

#### 8.3.1 Peer-support networks

To mitigate the identified “social isolation”, correctional institutions should formalize “Social Capital” through structured peer-mentorship programs. These programs should provide a safe space for teachers to share coping mechanisms and emotional burdens, reducing the psychological toll of the correctional environment.

#### 8.3.2 Resilience-building wellness programs

Actionable steps include providing access to confidential counseling and resilience workshops that aim to promote educators' wellness.

### 8.4 Policy reform

Policymakers should revise administrative frameworks to grant educators greater professional autonomy and dedicated time for “self-care practices” during the workday. Reducing “structural and institutional constraints” through clear protocols for educator-security collaboration can empower teachers and enhance their sense of self-efficacy.

## Data Availability

The original contributions presented in the study are included in the article/Supplementary material, further inquiries can be directed to the corresponding author.
