# Supplementary material for: Psychological well-being and resilience experiences of educators teaching in juvenile correctional facilities: a scoping review
Source: Front Psychol. 2026 Jan 13;16:1743604. doi: 10.3389/fpsyg.2025.1743604 (PMC12834796; doi:10.3389/fpsyg.2025.1743604)
Supplement: Supplementary file 1 [file Data_Sheet_1.pdf]

Appendix A: Search strategy among targeted databases

Targeted databases: ERIC, PsycINFO, Criminal Justice Abstracts with Full Text, Academic Search Complete, JSTOR, Google Scholar, Scopus and Web of Science

Construct synonyms

Population – (educator* OR teacher* OR instructor* OR tutor* OR trainer*)

Concept – (experiences OR “psychological well-being” OR “mental health” OR “emotional well-being” OR “emotional resilience” OR “psychological health” OR “mental wellness” OR "well being" OR wellbeing)

Context – (juvenile correctional education OR juvenile correctional facilit* OR juvenile detention center* OR youth correctional facilit* OR youth detention centre* OR juvenile prison* OR juvenile justice centre* OR youth justice center*) (*Setting*)

Search parameters - (*human participants*)

Search strategies used

(educator* OR teacher* OR instructor* OR tutor* OR trainer*) AND (experiences OR psychological well-being OR mental health OR emotional well-being OR emotional resilience OR psychological health OR mental wellness OR "well being" OR wellbeing) AND (juvenile correctional education OR juvenile correctional facilit* OR juvenile detention center* OR youth correctional facilit* OR youth detention centre* OR juvenile prison* OR juvenile justice centre* OR youth justice center*)

(educator* OR teacher* OR instructor* OR tutor* OR trainer*) AND (experiences OR psychological OR “well being” OR wellbeing OR mental OR emotional) AND (juvenile OR youth) AND (correctional OR detention OR prison* OR justice)

(educator* OR teacher* OR instructor* OR tutor* OR trainer*) AND (experiences OR psychological OR “well being” OR wellbeing OR mental OR emotional) AND (juvenile OR youth) AND incarcerated

ERIC (Education Resources Information Center) on EBSCOHost

TI (educator* OR teacher* OR instructor* OR tutor* OR trainer*) AND (experiences OR psychological well-being OR mental health OR emotional well-being OR emotional resilience OR psychological health OR mental wellness OR "well being" OR wellbeing) AND TI (juvenile correctional education OR juvenile correctional facilit* OR juvenile detention center* OR youth correctional facilit* OR youth detention centre* OR juvenile prison* OR juvenile justice centre* OR youth justice center*)

TI (educator* OR teacher* OR instructor* OR tutor* OR trainer*) AND TI (experiences OR psychological OR “well being” OR wellbeing OR mental OR emotional) AND TI (juvenile OR youth) AND TI (correctional OR detention OR prison*)

TI (educator* OR teacher* OR instructor* OR tutor* OR trainer*) AND (experiences OR psychological OR “well being” OR wellbeing OR mental OR emotional) AND (juvenile OR youth) AND incarcerated

APA PsycINFO

TI (educator* OR teacher* OR instructor* OR tutor* OR trainer*) AND (experiences OR psychological well-being OR mental health OR emotional well-being OR emotional resilience OR psychological health OR mental wellness OR "well being" OR wellbeing) AND TI (juvenile correctional education OR

juvenile correctional facilit* OR juvenile detention center* OR youth correctional facilit* OR youth detention centre* OR juvenile prison* OR juvenile justice centre* OR youth justice center*)

TI (educator* OR teacher* OR instructor* OR tutor* OR trainer*) AND TI ((experiences OR psychological OR “well being” OR wellbeing OR mental OR emotional) AND TI (juvenile OR youth) AND TI (correctional OR detention OR prison*)

TI (educator* OR teacher* OR instructor* OR tutor* OR trainer*) AND (experiences OR psychological OR “well being” OR wellbeing OR mental OR emotional) AND (juvenile OR youth) AND incarcerated

ProQuest Criminology Collection

title(educator* OR teacher* OR instructor* OR tutor* OR trainer*) AND (experiences OR psychological well-being OR mental health OR emotional well-being OR emotional resilience OR psychological health OR mental wellness OR "well being" OR wellbeing) AND title(juvenile correctional education OR juvenile correctional facilit* OR juvenile detention center* OR youth correctional facilit* OR youth detention centre* OR juvenile prison* OR juvenile justice centre* OR youth justice center*)

title(educator* OR teacher* OR instructor* OR tutor* OR trainer*) AND title(experiences OR psychological OR "well being" OR wellbeing OR mental OR emotional) AND (juvenile OR youth) AND (correctional OR detention OR prison*)

title(educator* OR teacher* OR instructor* OR tutor* OR trainer*) AND title(experiences OR psychological OR "well being" OR wellbeing OR mental OR emotional) AND (juvenile OR youth) AND incarcerated

Academic Search Ultimate

TI (educator* OR teacher* OR instructor* OR tutor* OR trainer*) AND (experiences OR psychological well-being OR mental health OR emotional well-being OR emotional resilience OR psychological health OR mental wellness OR "well being" OR wellbeing) AND (juvenile correctional education OR juvenile correctional facilit* OR juvenile detention center* OR youth correctional facilit* OR youth detention centre* OR juvenile prison* OR juvenile justice centre* OR youth justice center*)

TI (educator* OR teacher* OR instructor* OR tutor* OR trainer*) AND TI ((experiences OR psychological OR “well being” OR wellbeing OR mental OR emotional) AND TI (juvenile OR youth) AND TI (correctional OR detention OR prison*)

TI (educator* OR teacher* OR instructor* OR tutor* OR trainer*) AND (experiences OR psychological OR “well being” OR wellbeing OR mental OR emotional) AND (juvenile OR youth) AND incarcerated

APA PsycARTICLES

(educator* OR teacher* OR instructor* OR tutor* OR trainer*) AND (experiences OR psychological well-being OR mental health OR emotional well-being OR emotional resilience OR psychological health OR mental wellness OR "well being" OR wellbeing) AND (juvenile correctional education OR juvenile correctional facilit* OR juvenile detention center* OR youth correctional facilit* OR youth detention centre* OR juvenile prison* OR juvenile justice centre* OR youth justice center*)

(educator* OR teacher* OR instructor* OR tutor* OR trainer*) AND (experiences OR psychological OR mental OR emotional OR emotional OR "well being" OR wellbeing) AND (juvenile OR youth) AND (correctional OR detention OR incarcerated OR prison*)

Education Source

TI (educator* OR teacher* OR instructor* OR tutor* OR trainer*) AND (experiences OR psychological well-being OR mental health OR emotional well-being OR emotional resilience OR psychological health OR mental wellness OR "well being" OR wellbeing) AND TI (juvenile correctional education OR juvenile correctional facilit* OR juvenile detention center* OR youth correctional facilit* OR youth detention centre* OR juvenile prison* OR juvenile justice centre* OR youth justice center*)

TI (educator* OR teacher* OR instructor* OR tutor* OR trainer*) AND TI ((experiences OR psychological OR “well being” OR wellbeing OR mental OR emotional) AND TI (juvenile OR youth) AND TI (correctional OR detention OR prison*

TI (educator* OR teacher* OR instructor* OR tutor* OR trainer*) AND (experiences OR psychological OR “well being” OR wellbeing OR mental OR emotional) AND (juvenile OR youth) AND incarcerated

JSTOR

(ti:"educator* OR teacher* OR instructor* OR tutor* OR trainer*") AND (ti:"experiences OR psychological OR well being OR wellbeing OR mental OR emotional")) AND (ti:"juvenile OR youth")) AND (ti:"correctional OR detention OR prison* OR justice")

(ti:"educator* OR teacher* OR instructor* OR tutor* OR trainer*") AND (experiences OR psychological OR “well being” OR wellbeing OR mental OR emotional)) AND (juvenile OR youth)) AND (incarcerated))

Scopus

(educator* OR teacher* OR instructor* OR tutor* OR trainer*) AND (experiences OR "psychological well-being" OR "mental health" OR "emotional well-being" OR "emotional resilience" OR "psychological health" OR "mental wellness" OR "well being" OR wellbeing) AND ("juvenile correctional education" OR "juvenile correctional facilit*" OR "juvenile detention center*" OR "youth correctional facilit*" OR "youth detention centre*" OR "juvenile prison*" OR "juvenile justice centre*" OR "youth justice center*")

Ti: (educator* OR teacher* OR instructor* OR tutor* OR trainer*) AND Ti: (experiences OR psychological OR "well being" OR wellbeing OR mental OR emotional) AND Ti: (juvenile OR youth) AND Ti: (correctional OR detention OR prison OR justice) ??

Ti: (educator* OR teacher* OR instructor* OR tutor* OR trainer*) AND ti: (experiences OR psychological OR “well being” OR wellbeing OR mental OR emotional) AND (juvenile OR youth) AND incarcerated

Web of Science Core Collection (all citation indexes)

Ti: (educator* OR teacher* OR instructor* OR tutor* OR trainer*) AND ti: (experiences OR psychological well-being OR mental health OR emotional well-being OR emotional resilience OR psychological health OR mental wellness OR "well being" OR wellbeing) AND ti: (juvenile correctional education OR juvenile correctional facilit* OR juvenile detention center* OR youth correctional facilit* OR youth detention centre* OR juvenile prison* OR juvenile justice centre* OR youth justice center*)

Ti: (educator OR teacher OR instructor OR tutor OR trainer) AND ti: (experiences OR psychological OR “well being” OR wellbeing OR mental OR emotional) AND ti: (juvenile OR youth) AND ti: (correctional OR detention OR prison OR justice)

Ti: (educator* OR teacher* OR instructor* OR tutor* OR trainer*) AND (experiences OR psychological OR “well being” OR wellbeing OR mental OR emotional) AND (juvenile OR youth) AND incarcerated

Google Scholar

(educators OR teachers OR instructors OR tutors OR trainers) AND (experiences OR "psychological well-being" OR "mental health" OR "emotional well-being" OR "emotional resilience" OR "psychological health" OR "mental wellness" OR "well being" OR wellbeing) AND ("juvenile correctional education" OR "juvenile correctional facilities" OR "juvenile detention center" OR "youth correctional facility" OR "youth detention centre" OR "juvenile prison" OR "juvenile justice centre" OR "youth justice center")

allintitle: (educators OR teachers OR instructors OR tutors OR trainers) AND (experiences OR psychological OR “well being” OR wellbeing OR mental OR emotional) AND (juvenile OR youth) AND (correctional OR detention OR prisons OR justice)

(educators OR teachers OR instructors OR tutors OR trainers) AND (experiences OR psychological OR “well being” OR wellbeing OR mental OR emotional) AND (juvenile OR youth) AND incarcerated
